# An Overview of the Environmental Applicability of Vermicompost: From Wastewater Treatment to the Development of Sensitive Analytical Methods

**DOI:** 10.1155/2014/917348

**Published:** 2014-01-21

**Authors:** Madson de Godoi Pereira, Lourdes Cardoso de Souza Neta, Maurício Paulo Ferreira Fontes, Adriana Nascimento Souza, Thaionara Carvalho Matos, Raquel de Lima Sachdev, Arnaud Victor dos Santos, Marluce Oliveira da Guarda Souza, Marta Valéria Almeida Santana de Andrade, Gabriela Marinho Maciel Paulo, Joselito Nardy Ribeiro, Araceli Verónica Flores Nardy Ribeiro

**Affiliations:** ^1^Departamento de Ciências Exatas e da Terra, Universidade do Estado da Bahia, Rua Silveira Martins, 2555, 41.150-000 Salvador, BA, Brazil; ^2^Departamento de Solos, Universidade Federal de Viçosa, Avenida P. H. Rolphs, S/N, 36.570-000 Viçosa, MG, Brazil; ^3^Centro de Ciências da Saúde, Universidade Federal do Espírito Santo, Avenida Maruípe, S/N, Maruípe, 29.042-751 Vitória, ES, Brazil; ^4^Coordenação de Licenciaturas, Instituto Federal de Educação, Ciência e Tecnologia do Espírito Santo, Avenida Vitória, 1729, Jucutuquara, 29.040-780 Vitória, ES, Brazil

## Abstract

The use of vermicompost (humified material) for treating wastewaters, remediating polluted soils, improving agricultural productivity, protecting crop production, and developing sensitive analytical methods is reviewed here, covering the past 17 years. The main advantages of vermicompost, considering all applications covered in this paper, comprise (i) easy acquisition, (ii) low costs, (iii) structural, chemical, and biological characteristics responsible for exceptional adsorptive capacities as well as pollutant degradation, and (iv) the promotion of biocontrol. Specifically, for wastewater decontamination, a considerable number of works have verified the adsorption of toxic metals, but the application of vermicompost is still scarce for the retention of organic compounds. Problems related to the final disposal of enriched vermicompost (after treatment steps) are often found, in spite of some successful destinations such as organic fertilizer. For decontaminating soils, the use of vermicompost is quite scarce, mainly for inorganic pollutants. In agricultural productivity and biocontrol, vermicompost imparts remarkable benefits regarding soil aggregation, plant nutrition, and the development of beneficial microorganisms against phytopathogens. Finally, the use of vermicompost in sensitive analytical methods for quantifying toxic metals is the newest application of this adsorbent.

## 1. Humified Matter, Vermicompost, and Vermicomposting Processes

Humified matter comprises one the most important classes of natural chemical substances, whose origin is predominately derived from the decomposition of plant issues. The very intricate humic structures are produced after chemical and/or microbiological modifications of proteins, carbohydrates, nucleic acids, and lipids, as well as more complex compounds such as lignin and cellulose [1].

In the humification processes, biopolymers are broken into small fragments and randomly combined into humic precursors with different sizes and degrees of aromaticity. Given that a long period of time and adequate conditions of humidity and temperature are provided, drastic structural modifications occur and three main classes of humic substances are produced: humines, humic acids, and fulvic acids [1]. Humines have high contents of carbon and weak solubility in aqueous media, independent of the hydrogen ionic concentration. On the other hand, fulvic acids (C_12_H_12_O_9_N, minimum formula disregarding sulfur) have a higher percentage of oxygen and excellent solubility even in very acidic media. Due to their intermediary structural and chemical properties, humic acids (C_10_H_12_O_5_N as minimum formula) are extracted in diluted alkali solutions and present poor solubility in diluted acid [2–4].

It is important to note that each category of humic substance (humines and humic or fulvic acids) cannot be considered as a group containing molecules of a same compound. Instead, each one of these three categories presents thousands of different compounds but with similar solubility properties. Due to the extraordinary structural complexity of humic compounds it is highly unlikely to find two identical molecules of humine, fulvic, or humic acids in any natural environment [2].

According to Stevenson [2], the molecular weights of humic substances range from 500 to 5,000 Da for fulvic acids and from 3,000 to 1,000,000 Da for humic acids. To contribute to the structural elucidation of humic substances, Kucerik et al. [5] performed thermogravimetric analyses of diverse humic substances and found different profiles of combustion, which are compatible with the structural complexity reported by Stevenson [2].

Natural humification is very slow and, because of this, the production of humic matter is frequently accelerated by composting organic residues [6]. Imbeah [7] described composting as all processes which recycle agricultural and municipal wastes in order to produce matrices able to restore nutrients and increase soil fertility. According to Imbeah [7], composting is an environmentally acceptable method for treating wastes and its product furnishes the following benefits when compared with raw wastes: (i) pathogens are destroyed, (ii) nitrogen is converted into stable organic forms, (iii) the volume of wastes is considerably decreased, and (iv) the general physical-chemical characteristics of residues are improved.

In a complementary classification of composting, Fornes et al. [8] defined this method as any procedure in which indigenous microorganisms (thermophiles and mesophiles) transform organic compounds by means of aerobic processes. During the thermophilic phase (45 to 70°C) of composting, the sanitization of organic matter is observed, while the mesophilic phase is responsible for the maturation of diverse recalcitrant compounds. Maturation occurs similarly to humification, verified in soils when the phytotxicity of different chemicals is largely reduced [7].

Besides composting, vermicomposting comprises an attractive option as materials very rich in humified compounds can be produced in shorter periods of time. Controversial to the composting, vermicomposting uses earthworms for ingesting and metabolizing organic detritus in a complex mechanism composed of the following steps: (i) softening of residues by the saliva in the mouth of these animals; (ii) neutralization by calcium excreted from the inner walls of the esophagus; (iii) grinding of particles in the muscular gizzard; (iv) digestion of organic mass by proteolytic enzymes contained in the stomach, and (v) decomposition of pulped material by the action of many enzymes such as proteases, amylases, and lipases, among others [6, 8]. After this intensive biochemical activity, the vermicompost is excreted and continuously matured for a period up to six months in order to increase the contents of humified compounds and achieve additional stabilization of organic matter [8, 9].

**Figure 1:**
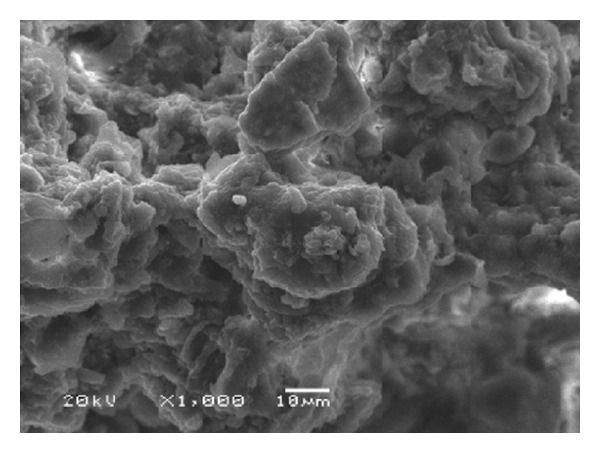
Electron micrograph of a vermicompost sample (1,000x).

**Figure 2:**
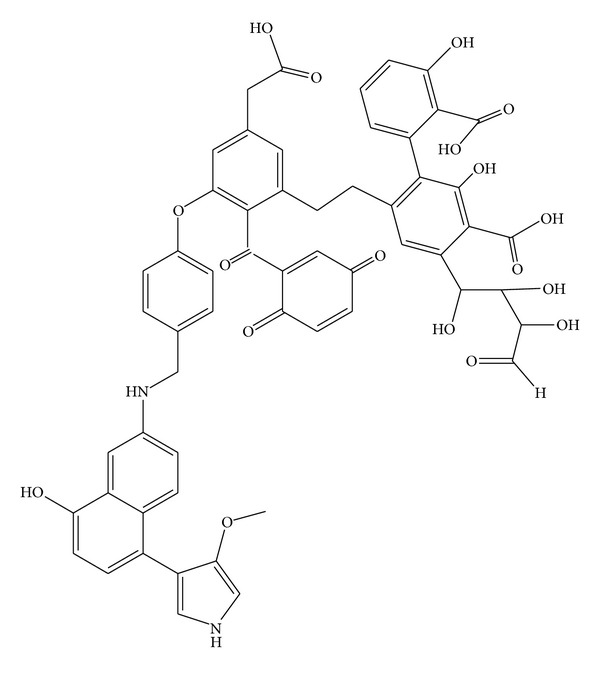
Structural fragment of humic acid [4].

As with any humified material, vermicompost presents numerous hydrophilic groups (–OH, –COOH, and –SH, among others), a high surface area, and vast porosity [6, 9, 10], thus demonstrating its remarkable adsorptive potential.  Figure 1 highlights the porous nature of vermicompost, while Figure 2 shows a fragment of humic acid (a macroconstituent of vermicomposts) with numerous chemical groups.

**Figure 3:**
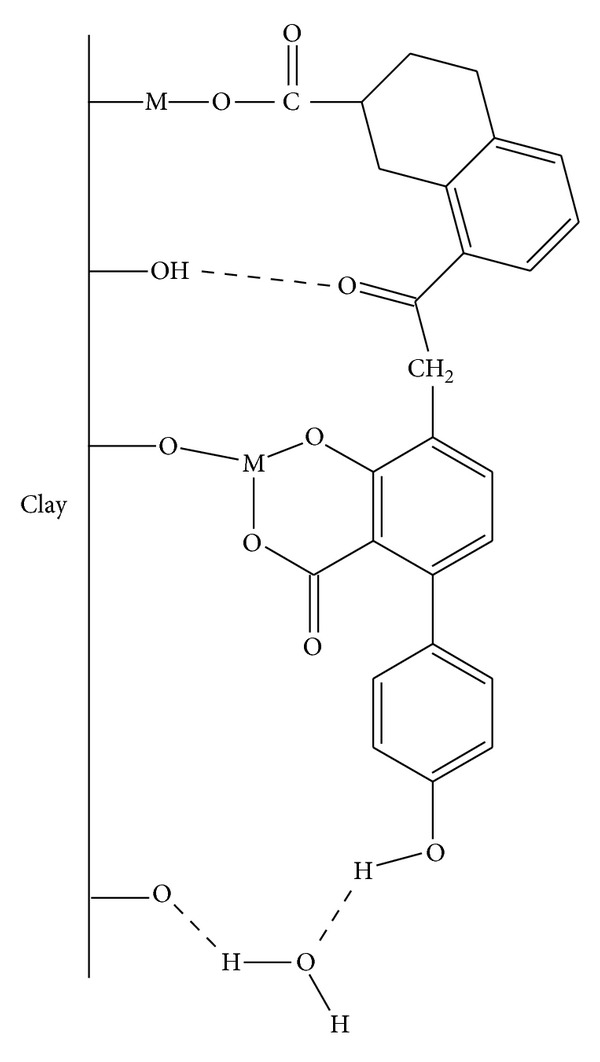
Interaction between humic substances and clays [4].

Because of the elevated contents of argillaceous minerals in vermicomposts, humic molecules frequently interact with surface of clays and form complex and stable structures, as indicated in Figure 3. This fixation, which is also found in soils and sediments, changes both the structure of humic compounds as well as that of clays [4].

Once humic substances are stabilized and vermicomposting increases/accelerates humification, it is possible to classify the production of vermicompost as an efficient way for stabilizing organic residues. In this sense, Romero et al. [11] verified the extensive maturation of cattle manure after vermicomposting, which promoted notable decreases in hydrogen concentrations and quantities of aliphatic structures as well as polypeptidic and carbohydrate components. Conversely, oxygen levels and the amount of acidic functional groups were considerably increased. As a consequence of this, organic residues achieved adequate maturity and stability as well as considerable enrichment of the humic acid fraction.

Lv et al. [12] demonstrated that water extractable organic matter (WEOM) was transformed during vermicomposting of cattle dung. The authors observed decreases in the concentrations of aliphatic compounds, proteins, and carbohydrates, with a concomitant elevation in aromaticity and oxygen-containing functional groups. In an analogous study, Subramanian et al. [13] evaluated the effects of composting and vermicomposting on sago wastes and observed many advantages of vermicomposting in order to produce valuable manure with good nutritional properties (in terms of C : N ratio) and higher contents of humic matter.

Lléo et al. [14] also verified that vermicomposting was the best option for treating the organic fraction of municipal solid waste, when compared with traditional composting procedures. The conclusions were based on the lower emission of pollutant gases such as CH_4_ and NH_3_, among other volatile compounds, during the production of vermicompost. In the same way, industrial solid wastes from a textile factory were suitably stabilized by means of vermicomposting with the production of an organic material with good properties for agricultural use [15].

Because vermicomposting increases the humic acid content and emits smaller quantities of atmospheric pollutants, this procedure can be considered more advantageous than composting. In addition to these two characteristics, Nagavallemma et al. [16] showed that vermicompost accumulates higher levels of macro- and micronutrients (organic C, N, P, K, Ca, Mg, Na, Zn, Cu, Fe, and Mn) when compared to garden compost. Moreover, it was demonstrated that some plant nutrients (nitrogen and phosphorus, for example) exhibited greater availability when vermicomposts were applied to soils [17].

Since vermicomposting presents many advantages, this practice has been employed as a valuable tool for treating large quantities of wastes (from municipal and other sources) in populous countries such as India, Malaysia, and China [15, 18–22]. However, the presence of harmful metals and microorganisms should always be checked, mainly when this humic substrate is employed as an organic fertilizer. Related to this subject, Azizi et al. [23] demonstrated the great capacity of earthworms to assimilate heavy metals from the environment, thus underlining the real possibility of the contamination of vermicompost if raw materials containing high contents of pollutants are used.

In terms of processing, vermicomposting can be performed by means of simple procedures, in which a great variety of organic matter derived from agricultural [24], industrial [25], or domestic sources [26] is mixed with earthworms. Additionally, different kinds of soils are often added to organic substrates, and this procedure is responsible for the presence of high contents of quartz, feldspar, and kaolinite, among other minerals in the vermicompost composition [27].

According to Nagavallemma et al. [16], vermicomposting is habitually performed in pits below the ground, but heaping on the soil has also been successfully adopted. Alternatively, vermicomposting is performed in tanks made up of different materials such as bricks, hollow bricks, asbestos, local rocks, and cement rings above the soil, among others. In general, the best period for releasing earthworms into organic residues is between 15 and 20 days after heaping, when temperatures are near 25°C [16].

Garg et al. [28] employed *Eisenia foetida *to produce vermicomposts from different types of organic residues, including kitchen wastes, agroresidues, institutional and industrial wastes, textile industry sludge, and fibers. According to the authors, some differences were observed among vermicomposts, including the contents of phosphorus, potassium, and nitrogen as well as total organic carbon. Pereira and Arruda [6] also verified that vermicomposts derived from different organic residues presented large variations in the content of sulfur and nitrogen. Notwithstanding the importance of correlations between the chemical composition of vermicompost and its raw material, there have been few studies concerning this subject. Further investigations are crucial to obtain matrices with better adsorptive selectivity, for example.

## 2. Environmental Applications of Vermicompost

### 2.1. Vermicompost for Treating Aqueous Media

In the literature, vermicompost has been reported as an efficient adsorbent for toxic or potentially toxic metals and organic pollutants contained in aqueous media. In general, experimental designs consider the optimization of parameters, such as pH, mechanical agitation time, column flow rates, particle size, and adsorbent mass. Additionally, techniques based on thermogravimetry, infrared spectrophotometry, X-ray diffractometry, and electron microscopy are often employed to elucidate the structural and chemical aspects of vermicompost. This characterization is necessary, because vermicomposts are very heterogeneous materials and there are no guaranties regarding uniformity in terms of chemical composition.  Table 1 lists the diverse uses of vermicompost in wastewater decontamination, while the following paragraphs contain a discussion about the relevant aspects of some cited manuscripts.

Some studies listed in Table 1 present limitations regarding the saturation of vermicompost, which exhibits a great number of superficial negative charges responsible for the unspecific retention of cations. Thus, aqueous media containing high ionic concentrations can be problematic if some procedures, such as those related to increasing vermicompost mass, are not performed.

Jordão et al. [29] filled glass columns (38 cm long and 7 cm i.d.) with adequate quantities of vermicompost and achieved the complete removal of Cd^2+^, Cu^2+^, chromium (as Cr_2_O_7_^2−^), Ni^2+^, and Zn^2+^ from galvanoplastic effluents and/or synthetic aqueous solutions. For aqueous solutions, the following values of retention were observed: 6,000 mg kg^−1^ for Cu^2+^ and Ni^2+^ and 4,000 mg kg^−1^ for Cd^2+^ and Zn^2+^. In galvanoplastic effluents, retentions of 2,000 mg kg^−1^ (Cr_2_O_7_^2−^ and Zn^2+^) and 4,000 mg kg^−1^ (Ni^2+^) were obtained. The vermicompost mass employed to retain metallic cations from synthetic solutions was also enough to treat galvanoplastic effluents, thus reinforcing the notion that adequate choices regarding vermicompost mass solve limitations related to the complexity of the matrix. An additional advantage found by Jordão et al. [29] associated with large masses of vermicompost was the increase in pH (from 2 to approximately 6) in all treated media. The decline in acidity was due to the sequestration of H_3_O^+^ by chemical groups in vermicompost; this property is very advantageous from an environmental point of view, because many effluents containing metallic cations are acidic.

When Jordão et al. [29] amended different tropical soils with enriched vermicompost, they observed adequate transfer of essential elements to plants. Nevertheless, prohibitive amounts of cadmium were also found, and this result reveals that constant evaluation is necessary in order to guarantee safe disposal of enriched vermicompost in soils. In this case, the assimilation of metals by plants depended on the soil properties, total organic content of the vermicompost, species, and age of the plants, as well as climate.

Jordão et al. [30] employed vermicompost in a column system for treating galvanoplastic effluents, and it was observed that copper, nickel, and zinc ions were retained with efficiencies close to 100% at natural effluent pH: 2.0, 7.4, and 6.9 for Cu^2+^, Ni^2+^, and Zn^2+^, respectively. When vermicompost was mixed to tropical soils, alarming transfers of metals to lettuce were not observed.

Matos and Arruda [33] treated effluents from a chemical laboratory with vermicompost, and they achieved excellent adsorption for Cd^2+^, Cu^2+^, and Pb^2+^ as well as Zn^2+^, similar to the work by Jordão et al. [29], in which the pH of the effluent was considerably increased during treatment. The enriched vermicompost was mixed with soil for the subsequent cultivation of sunflowers, and no biological damage was observed in the plants. However, studies on the concentration of cadmium and lead were not performed in the edible parts (oils and seeds) of the sunflowers. This would be of great importance if aspects related to food safety are considered.

Zaragosa et al. [34] studied different forms of the interaction between vermicompost and Pb^2+^, and they concluded that the retention of lead ions was performed by means of covalent bonds and electrostatic attractive forces as well as precipitation and coprecipitation; these two last processes attributed to the association of Pb^2+^ with some anions in vermicompost (phosphates). This multiple association of Pb^2+^ with different structural components of vermicompost explains the considerable retention of this metallic cation, as already reported for vermicomposts from different sources [30, 41, 43].

Jordão et al. [35] used vermicompost to remove Al^3+^ and Fe^2+^ from synthetic aqueous solutions and industrial wastewaters derived from a mineral processing unit. After 4 h of a batch procedure, very quantitative retentions (~100%) were observed for both metallic ions at pH 2, thus reinforcing the idea that the use of an appropriate mass of vermicompost is able to supplant all undesirable effects concerning high acidity. Probably, aluminum was also removed by precipitation of Al(OH)_3_, since vermicomposts promote considerable increases in the pH of aqueous solutions, as previously reported [29, 33].

Similar to metallic cations, organic pollutants can be removed from aqueous media by employing vermicompost, as demonstrated by Pereira et al. [36], who used vermicompost for decoloring aqueous solutions contaminated with two cationic dyes: crystal violet and methylene blue. For this, glass columns were filled with 40 g of the adsorbent and percolated with single dye solutions (20 mg L^−1^), at flow rates of 5 or 20 mL min^−1^. After treating at least 5 L of solution, 40 g of used vermicompost was incinerated and the inert ashes (oxides, mainly) were obtained. Tests performed in batch assemblies indicated fast chemical equilibrium, thus evidencing unspecific and reversible adsorption of both molecular species. Due to the large spatial size of both dyes, the maximum adsorptive capacities were smaller than those reported for other natural adsorbents.

The adsorption capacity of vermicompost for organic chemicals was also investigated by Mendes et al. [37], who evaluated the retention of a pesticide (methylparathion). Under controlled experimental conditions of pH (6.8) and agitation time (61.5 minutes), an excellent maximum adsorptive capacity of 170 mg kg^−1^ was achieved under conditions of irreversibility, which was confirmed by elution tests with an HNO_3_ solution at pH 3.0. After elution, detectable quantities of methylparathion were not observed, even when using a sensitive voltammetric technique. Another indication of irreversibility was the relatively long equilibrium time (61.5 minutes), which is typical of chemical adsorption with a notable degree of covalent bonding.

As charged groups are numerous on the vermicompost surface, processes based on Coulomb forces tend to be proceeded very quickly. In contrast, specific and effective spatial orientations are necessary to establish adsorption based upon covalent bonds. As a consequence, longer periods of time are necessary for accomplishing chemical adsorptions.

According to the physical-chemical properties of the adsorbate, there are some possibilities of interaction with vermicompost. In this sense, electrostatic and reversible adsorptions are expected when inorganic or organic cations are considered. However, the great number of electrical charges in vermicompost does not ensure exclusive reversible retention, because cations of transition metals (with free energy levels) tend to form covalent bonds with diverse chemical groups [43]. As already discussed by Mendes et al. [37], adsorption with some irreversibility was verified for a neutral organic compound (methylparathion).

Lamim and collaborators showed mutual and similar decreases in the retention of Cu^2+^ and Zn^2+^ [38] as well as Cd^2+^ and Pb^2+^ [41] when each of these pairs was concomitantly adsorbed by vermicompost. In addition to a large excess of negative charges in the vermicompost structure, similarities between the densities of charge for Cu^2+^ and Zn^2+^ as well as Cd^2+^and Pb^2+^ can be pointed out as another reason for the predominance of unspecific adsorption.

Reversible adsorption on vermicompost permits recovery/reuse of pollutants contained in wastewaters. This perspective is very interesting, because production lines can be fed back with their own discharges. As an example, vermicompost enriched with metals from galvanoplastic wastes could be leached with the subsequent reintroduction of eluted metallic ions in saline baths employed for electrodeposition. Options like this should be explored in order to motivate sustainable processes of production as well as create new alternatives to avoid or diminish the use of enriched vermicomposts in soils. This concern is especially relevant when vermicomposts enhanced with very toxic metals (cadmium and lead, for example) are considered.

A very lethal pesticide (metribuzin) was efficiently adsorbed by vermicompost and degraded by the natural microbial population of the cited adsorbent [9]. This cycle of retention/degradation is greatly desirable, because it eliminates steps of disposal that can be slow and laborious. Due to the extensive biological and chemical variability of vermicomposts, it is not possible to assure microbial decomposition for all classes of organic pollutants, but this possibility is very exciting and should always be evaluated.

In an innovative way, Taylor et al. [39] employed vermicompost for removing soluble and biodegradable organic compounds from domestic wastewaters. During waste percolation, many physical-chemical parameters were monitored, and notable changes were observed in the waste composition. Preliminary results showed a reasonable reduction in the dissolved organic matter content, but a conclusive evaluation of vermicompost performance was not possible. In this specific case, the influence of suspended solid particles on the filtering capacity of vermicompost was not completely understood, and the necessity of additional investigations was pointed out. In spite of this, this study is very interesting because it contemplates a class of pollutants poorly considered in treatments based on adsorption. In general, biodegradable compounds are eliminated by biological technologies such as aerobic tanks, where bacteria promote efficient degradation under conditions of oxygen saturation and controlled pH. Probably, the natural microbial population of the vermicompost was responsible for decomposing part of the organic compounds found in the domestic wastewater.

The work of Carrasquero-Durán and Flores [42] is very interesting, because it employed an analytical technique (infrared spectroscopy) to identify structural modifications in vermicompost before and after the retention of Pb^2+^ at pH 3.8 and 7.0. The typical peak of carboxylate groups was observed at the highest pH, while a strong and wide peak between 1361 and 1388 cm^−1^ was attributed to chelated Pb^2+^. As previously demonstrated by other authors [30, 33, 41], Carrasquero-Durán and Flores [42] also shown that vermicompost presents expressive adsorptive capacities for lead, 113.6 and 123.5 mg g^−1^ at 33 and 50°C, respectively.

When vermicompost has been employed for decontaminating aqueous media, there is an evident predominance of research from South America. Probably, the low cost and ease of acquisition are responsible for the notable use of vermicompost for decontaminating wastewaters in undeveloped and emerging countries, where aquatic ecosystems have been constantly submitted to situations of environmental stress, mainly due to the emission of toxic metals [44–48].

### 2.2. Vermicompost for Remediating Polluted Soils

The use of vermicompost for extracting or immobilizing metal(loid)s from soils is scarcely reported, but there are interesting studies dealing with the use of this humified adsorbent to decrease the impact of different organic pollutants. When vermicompost is added to polluted soils, different results can be obtained in relation to the mobility of pollutants. A great number of variables should be considered, such as the size of soil particles, the structural characteristics of the clays present in the soil, and the quantity and quality of the humic fraction in the vermicompost as well as the chemical and physical features of the pollutants. The studies described below investigated the use of vermicompost in polluted soils in order to decrease the levels and mobility of dangerous chemicals.

Álvarez-Bernal et al. [49] added vermicompost to soils to remove polycyclic aromatic hydrocarbons (PAHs), but unsatisfactory results were achieved. As a consequence, high residual quantities of phenanthrene, anthracene, and benzo*(a)*pyrene were found in the evaluated soils. Similarly, Contreras-Ramos et al. [50] verified the inefficiency of vermicompost in extracting the same PAHs from other polluted soils. In the latter study, earthworms and biosolids were also employed, and the vermicompost exhibited the lowest effectiveness in terms of extraction. This low efficiency could be attributed to the weak polarity of PAHs, which minimizes or nullifies the thermodynamic tendency related to the transfer of these organic pollutants to vermicompost that presents high quantities of electrical charges.

On the other hand, vermicomposts were successfully used to reduce the availability of an herbicide [3-(3,4-dichlorophenyl)-1,1-dimethylurea, or diuron] in soils [51]. As diuron (C_9_H_10_Cl_2_N_2_O) exhibits reasonable polarity, there is considerable affinity between this compound and the hydrophilic groups of vermicompost, with the consequent distribution of diuron through different horizons of amended soils. In a similar way, Fernández-Bayo et al. [52] studied the influence of vermicompost on the mobility of imidacloprid (C_9_H_10_ClN_5_O_2_) insecticide in many Spanish soils. As expected from considerations of polarity, vermicompost was responsible for substantial retention of imidacloprid, as observed for diuron [51].

Delgado-Moreno and Peña [53] added vermicompost derived from olive cake to soils contaminated with herbicides. In order to perform these experiments, calcareous soils were mixed with vermicompost and other substrates at rates four times higher than the recommended agronomic dose. It was observed that biological degradation increased during the first week of incubation, but residual concentrations of all herbicides (simazine, terbuthylazine, cyanazine, and prometryn) were similar between the nonamended and amended soils. In this sense, vermicompost increased the kinetics of herbicide decomposition (by means of microbial biostimulation), but it did not act on the thermodynamic aspect. Kadian et al. [54] observed that different pesticides had their concentrations diminished in soils after treatment with vermicompost. Again, the ability of vermicompost in stimulating microorganisms was pointed out as the cause of pesticide decomposition. Thus, the studies by Delgado-Moreno and Peña [53] and Kadian et al. [54] reinforce the idea that vermicompost improves the biological activity of soils, thus indicating the potential of vermicompost in bioremediation. According to Iwamoto and Nasu [55], the ability of organic amendments in biostimulating microorganisms is one the most important criteria in order to establish bioremediation in soils.

Vermicompost cannot only be considered as a source of microorganisms to soils but also as a supply of nutrients for the native microbiota of these ecosystems. From this perspective, it was observed that soils polluted with different herbicides [53, 56] had their microbial populations restored after the addition of vermicompost. In these specific cases, the greater part of the microbiota from the vermicompost was fixed to the soil.

In order to minimize the pollution of soils by heavy metals, Jordão et al. [57] added vermicompost to tropical soils with the intention of decreasing the mobility of Cd^2+^ and Cu^2+^. The authors achieved satisfactory results, which can be explained by accentuated spontaneity related to adsorptive processes (ΔG around −14,000 kJ mol^−1^). The work of Jordão et al. [57] shows that vermicompost is able to bioremediate soils containing metallic species and this ability is also extended to other organic substrates [58]. According to Park et al. [59], bioremediation of metals by organic substrates is a consequence of the following mechanisms: (1) immobilization, (2) reduction, (3) volatilization, and/or (4) modification of the rhizosphere.

In terms of immobilization, organic substrates (such as vermicompost) retain ionic species by means of adsorptive mechanisms, as already discussed. Probably, this was the most important mechanism in the bioremediation of Cd^2+^ and Cu^2+^, as observed by Jordão et al. [57]. Bioremediation based on reduction is possible, because vermicomposts provide a source of electrons and carbon for reducing microorganisms. On the other hand, volatilization is possible due to microbiological methylation of a restricted group of elements: As, Hg, and Se. Since microorganisms play an essential role in both reduction and methylation reactions and vermicompost provides substantial microbiota to soils and/or stimulates the native microbial population, the use of this humified substrate in soils is very promising for bioremediation based on reduction and methylation processes [59].

Finally, vermicomposts and diverse other organic amendments release weak acids (citric, maleic, lactic, oxalic, propanoic, and butyric acids, among others) to soils. This reduction in soil pH has profound consequences on the chemistry and biology of these ecosystems, especially in the rhizosphere that comprises the area immediately around the roots [58]. In this case, bioremediation is improved by excess of H_3_O^+^ in soil solution which stimulates the transfer of metallic pollutants to plants. This situation is clearly desirable only for plants devoted to removal of hazardous metals from polluted soils.

### 2.3. Vermicompost for Increasing Agricultural Productivity

Table 2 lists studies concerning the increase in agricultural productivity as a result of adding vermicomposts to soils. These mixtures have been pointed out as being responsible for increases in (i) seed germination due to increases in the temperature of soils (black color of the vermicompost); (ii) water retention promoted by the great number of hydrophilic groups in the structure of vermicompost; (iii) plant nutrient levels such as nitrogen, soluble potassium, exchangeable calcium, magnesium, and phosphorus, and (iv) the aggregation of particles [58]. Some studies cited in Table 2 are discussed below, but all of them had similar results independent of differences in climate, humidity, age, and species of plants as well as soil type.

After mixing vermicompost into soils used for cultivating tomato, Gutiérrez-Miceli et al. [60] observed excellent and progressive restoration of nutrients (nitrogen and metallic ions). This gradual transfer of essential elements was possible because humified materials present components with very good chemical stability and a long permanence time in soils [61]. In addition to the gradual release of nutrients, Albiach et al. [62] verified that vermicomposts with elevated levels of carbohydrates were able to stabilize particles of diverse soils. Thus, it was demonstrated that vermicomposts with a smaller degree of humification (or higher content of carbohydrates) can be as desirable as those in an advanced stage of maturation. This observation reinforces the notion that the use of adequate raw matter can produce vermicomposts with specific and desirable properties.

Singh et al. [74] performed an interesting study dealing with the foliar application of leachates obtained from vermicomposts (from cow dung, vegetable waste, and a mixture of them) in the cultivation of strawberry. The term leachate is applied to the liquid product of drainage from vermicomposting units, which is produced during aerobic oxidation of organic matter. As a result of leachate application, the authors observed increases in (i) leaf area, (ii) dry matter of plants, and (iii) fruit yield. The literature [74] reports that leachates of vermicomposts are excellent fertilizers containing high concentrations of plant nutrients and excellent levels of humic acids that regulate many processes related to the development of plants. In terms of plant nutrients, the leachates of vermicompost obtained by Singh et al. [74] presented calcium levels, varying from 71 to 94 mg L^−1^, while boron contents varied from 151 to 191 *μ*g L^−1^.

When fertilizers obtained from leachates of vermicompost were applied to cultures of sorghum, Gutiérrez-Miceli et al. [75] showed a notable increase in plant development after the establishment of a symbiotic association between fungi and the roots of sorghum. The leachates presented remarkable concentrations of potassium (834 mg L^−1^), nitrate (247 mg L^−1^), and phosphate (168 mg L^−1^), but these formulations were diluted in order to avoid toxicological damage to the plants. Because of this, it was necessary to employ additional sources of nutrients, such as synthetic NPK formulations. The use of excessive amounts of vermicompost always needs to be considered, because this substrate contains natural and accentuated levels of metallic species capable of damaging soil structure as well as contaminating plants and food chains.

Of the elements able to destroy the structure of soil, sodium should be emphasized, because high contents of this metal accelerate erosion and microbial death [78]. As previously discussed, the presence of toxic elements such as cadmium and lead in vermicompost is particularly worrying when this humified material is mixed with soils.

Different from the use of vermicompost for treating wastewaters, the application as agricultural fertilizer is largely performed in developed countries. This observation is justified by the strong social demands for sustainable agriculture and environmental protection. However, the limit of phytotoxicity associated with the large use of vermicompost in diverse soils is still a dubious aspect and needs to be investigated more closely.

The work of Atiyeh et al. [65] showed that excess of vermicompost was ineffective for the cultivation of tomatoes. In experiments performed in a greenhouse, appreciable vegetal growth was verified when the percentage of vermicompost in soil was in the range of 20 to 40% (m/m), but larger proportions of this humified material did not offer additional benefits. Probably, setbacks in agricultural production would be observed if the percentages of vermicompost were even higher, because excessive amounts of certain elements naturally found in vermicompost are toxic to plants. Such is the case of nitrogen that causes many biochemical and physiological disorders when accumulated as soluble proteins and aminoacids [79]. These excessive amounts of aminoacids also increase the vulnerability of plants to agricultural plagues, since palatability of vegetal issues is augmented for many herbivorous insects [80].

#### 2.3.1. Vermicompost in the Biocontrol of Agricultural Disease

**Figure 4:**
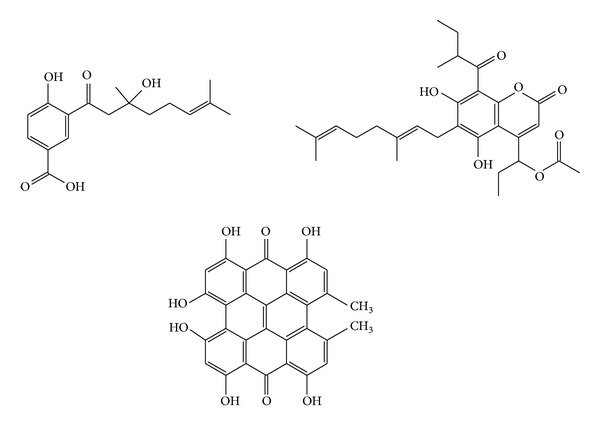
Examples of natural antifungals extracted from plants [77].

As previously discussed, when vermicomposts are mixed with soils, large amounts of nutrients are released for plants with a consequent increase in their natural defenses against disease [73, 81–83]. Additionally, vermicompost increases the biological resistance of plants by means of other mechanisms: (1) the stimulation of beneficial microorganisms in soils and the consequent protection of plants counter to phytopathogens [81], (2) the transfer from vermicompost to soil of microbial species with the ability to minimize or prevent damage caused by phytopathogens [16], (3) the presence of antibiotic and antifungal compounds in the vermicompost [16, 82], and (4) the induction of biosynthesis of antifungal compounds in plants [82]. Many antifungal compounds extracted from plants (Figure 4) have structures based on aromatic rings [77], thus justifying why vermicomposts with a high degree of humification (and aromaticity) are so important for stimulating the production of antifungal substances in vegetal organisms. Most are secondary metabolites, of which at least 13,000 have been isolated that is less than 10% of the total [77].

According to Pal and Gardner [84], the term biocontrol has been employed in different fields of biology, most notably in entomology and plant pathology. In the first field, the biocontrol comprises the use of live predatory insects, while in plant pathology it employs microbial antagonists to suppress diseases as well as host-specific pathogens to control weed populations. In this scope, the use of vermicompost can be classified as a form of biocontrol based on the field of phytopathology.  Table 3 lists the relevant studies on this subject.

In spite of the non-conclusive results, it was verified that substitution of an inorganic medium of cultivation by vermicompost was capable of decreasing infestation of agricultural plagues on different plants [80]. In this specific study, better results were obtained when substitution by vermicompost achieved values up to 40%. This experimental conclusion shows that insufficient doses of vermicompost limit the availability of some nutrients which are able to reduce the palatability of vegetal issues and, in consequence, plants become more attractive to many insects [80]. As previously discussed, large amounts of vermicompost in soils release excessive quantities of nitrogen and infestations by insects can increase. For this reason, careful studies should be performed in order to find ideal doses of this humified substrate.

Sahni et al. [82] evaluated the integrated use of vermicompost and an antagonistic strain of a bacterium in order to decrease the incidence of a disease called collar rot of chickpea (*Cicer arietinum*). The authors observed that the simultaneous use of vermicompost and *Pseudomonas syringae* resulted in a reduction in seedling mortality in chickpea under greenhouse conditions. Again, vermicompost augmented the nutrient levels in soils with an emphasis on iron, manganese, and phosphorus; this enrichment seemed to promote the development of *Pseudomonas syringae*.

In India, Singhai et al. [85] evaluated the effects of vermicompost against potato scab by testing the capacity of this humified material to stimulate the growth of four *Pseudomonas* strains. It was observed that vermicompost increased the growth of *Pseudomonas mosselii*, which promoted satisfactory plant growth allied with efficient defenses against phytopathogens.

Gopalakrishnan et al. [89] isolated cultures of actinomycetes from different herbal vermicomposts, and these cultures were tested against *Fusarium oxysporum* f. sp. *Ciceris* (FOC), a kind of fungus that causes several diseases in plants. An antagonist effect was verified for some actinomycete species counter to FOC, and this result was pointed out as being very promising in the search for ecofriendly bio-fungicides. This perspective is motivating, because it avoids the massive use of synthetic fertilizers/pesticides and the consequent toxicological effects on humans as well as terrestrial/aquatic environments [3, 4, 92, 93].

Edwards et al. [90, 91] showed that aqueous extracts from vermicomposts were capable of diminishing infestation of agricultural plagues on cucumbers and tomatoes. According to the authors, the presence of elevated concentrations of phenolic compounds in extracts was responsible for reducing pest reproduction and survival rates as well as decreasing the palatability of vegetal issues. The best effects related to biological defense against insects were achieved with the highest rate of aqueous extract application and deleterious effects caused by the excess of some elements, such as nitrogen, were not reported.

### 2.4. Utilization of Vermicompost in Environmental Analytical Chemistry

Because of its excellent cationic adsorptive capacity, vermicompost has been used to preconcentrate toxic metallic ions [94, 95]. This strategy is based on the accumulation of cations in vermicompost and the subsequent elution of them by passing reduced volumes of diluted acids, such as HCl and HNO_3_ [96]. This analytical proposal has a good environmental scope, because it can be directed to the quantification of pollutants at trace and ultratrace levels in an extensive number of samples, such as waters, foods, pharmaceuticals, and fuels, among others.

Pereira and Arruda [94] filled minicolumns with 25 mg of vermicompost in order to preconcentrate Cd^2+^ and Pb^2+^ with subsequent quantification of them by flame atomic absorption spectrometry (FAAS). In the cited work, preconcentration steps were performed for a flow injection analysis (FIA) system, in which 16.00 mL of aqueous samples was passed through minicolumns followed by elution with 220 *μ*L of an HNO_3_ solution at 3 mol L^−1^. The sensitivity of the FAAS technique was augmented by 46- and 62-fold for cadmium and lead, respectively. However, when real samples (digested foods and pharmaceuticals and biological matrices) were analyzed, considerable reductions in sensitivity were observed. This limitation was due to the lack of sensitivity attributed to vermicompost. The proposed methods [94] were satisfactorily operated only after employing the analyte addition method, which is able to circumvent effects of the matrix. An increase in vermicompost mass was tested in order to solve the problem of the selectivity of vermicompost (or the sensitivity of the analytical method), but this procedure resulted in many leaks through the connections of the FIA system.

Bianchin et al. [95] proposed the use of vermicompost for preconcentrating Cd^2+^ from samples of fuel alcohol with subsequent quantification of this analyte by FAAS. Again, a flow injection system was chosen, but a larger mass of vermicompost (160 mg) was employed when compared to the previously discussed study [94]. The proposed method was able to increase the sensitivity of FAAS by 32-fold considering a sample volume of 10.00 mL. In contrast to the previous work [94], serious problems related to selectivity were not observed and calibration by the analyte addition method was not necessary.

It is probable that the employment of vermicompost as a preconcentrator material in batch design would result in large preconcentration factors and poor selectivity could be solved to a great extent. Nevertheless, batch procedures involving vermicompost have not been performed and published since 2009, indicating that the structural, morphological, and chemical characteristics of vermicompost still cause detachments in applications as refined as those associated with analytical instrumentation. Although the pioneering works of Pereira et al. [94] and Bianchin et al. [95] showed the potential of vermicompost as a preconcentrator, and synthetic resins still seem to be more advantageous in terms of the figures of merit of analytical methods.

However, as already pointed in this review (Section 1) and reinforced by Campitelli and Ceppi [97], steps of vermicomposting can be controlled in order to produce vermicomposts with specific properties, including those related to increase of selectivity. This kind of investigation would result in remarkable improvements for applicability of vermicompost in analytical preconcentration.

## 3. Conclusions

During the last 17 years (the period covered by this review), vermicompost has been mainly used for (1) decontaminating aqueous media by adsorbing or degrading pollutants, (2) increasing soil fertility and agricultural productivity, (3) promoting the biocontrol of agricultural disease, and (4) raising the sensitivity of analytical methods on a minor scale. The first application is justified by alarming situations concerning aquatic pollution with the consequent necessity of alternative, efficient, and cost-effective procedures for purifying wastewaters. In this context, vermicompost is one the most important natural adsorbents, because it presents very low costs and good adsorptive capacities for a large variety of inorganic and organic pollutants. However, the disposal of enriched vermicompost (after treatment steps) still presents limitations and further research concerning the recovery of pollutants is necessary.

Successful environmental application of vermicompost has also been observed in agricultural practices since the addition of this humified material improves the physical-chemical properties of soils and the nutritional conditions of plants. For biocontrol proposals, vermicomposts have been successfully employed to suppress the growth of phytopathogenic microorganisms or to stimulate the development of those with beneficial biological characteristics. Moreover, some chemical constituents of vermicomposts promote the biosynthesis of natural compounds with remarkable antimicrobial activity.

Despite the promising results derived from the use of vermicompost as a preconcentrator of chemical species, this humified substrate is very limited by its poor selectivity. This undesirable feature is the main reason responsible for the small number of publications concerning the employment of vermicompost in analytical instrumentation devoted to the quantification of pollutants at trace and ultratrace levels.

From these conclusions, the following aspects related to vermicompost should be improved/understood: (i) the selectivity of vermicompost for circumventing fast saturations; (ii) recoveries of adsorbed species in order to establish procedures based on reuse and recycling, and (iii) the toxicity of large excess of vermicompost for plants cultivated in different kinds of soils.

## Figures and Tables

**Table 1:** Application of vermicompost for treating wastewaters.

Application	Retained chemical species	Country	Reference
Removal of metallic ion from aqueous solutions	Cd^2+^	Brazil	[6]
Decontamination of wastes and synthetic solutions	Cd^2+^, Cr^6+^, Cu^2+^, Pb^2+^, and Zn^2+^	Brazil	[29]
Decontamination of wastes	Cu^2+^, Ni^2+^, and Zn^2+^	Brazil	[30]
Decontamination of wastes	Zn^2+^	Brazil	[31]
Decontamination of wastes	Cr^3+^, Ni^2+^, Pb^2+^, and V^5+^	Argentina and Venezuela	[32]
Decontamination of wastes	Cd^2+^, Cu^2+^, Pb^2+^, and Zn^2+^	Brazil	[33]
Removal of metallic ion from aqueous media	Pb^2+^	Mexico	[34]
Removal of metallic ions from aqueous media	Al^3+^ and Fe^2+^	Brazil	[35]
Retention of organic dyes	Crystal violet and methylene blue	Brazil	[36]
Removal of pesticide from aqueous solutions	Methylparathion	Brazil	[37]
Removal of metallic ions from aqueous solutions	Cu^2+^ and Zn^2+^	Brazil	[38]
Removal of organic compounds from domestic wastes	Soluble organic compounds	Australia	[39]
Treatment of wastewater	Cd^2+^, Cu^2+^, Pb^2+^, and Zn^2+^	Czech Republic	[40]
Removal of metallic ions from aqueous solutions	Cd^2+^ and Pb^2+^	Brazil	[41]
Removal of metallic ions from aqueous solutions	Pb^2+^	Venezuela	[42]

**Table 2:** Applications of vermicompost to increase agricultural productivity.

Culture	Agricultural benefits	Country	Reference
Tomatoes (*Lycopersicum esculentum*)	Better plant growth and carbohydrate accumulation	Mexico	[60]
Maize	Improvements in growth	Mexico	[63]
Watercress (*Lepidium sativum*)	High productivity	Italy and Spain	[64]
Tomatoes (*Lycopersicon esculentum* Mill.)	Augmentation of growth	USA	[65]
Tomatoes	Soil fertility	Germany	[66]
Paddy	Higher nutrient availability	India	[67]
Peppers (*Capsicum annuum*)	Soil fertility	USA	[68]
Strawberry (*Fragaria ananasa*)	Soil fertility	USA	[69]
Greenhouse peppers (*Capsicum annum L. *var. California)	High growth of flowers and fruits	USA	[70]
*Pogostemon cablin* (patchouli) Benth	Improvements in soil properties and essential oil yield	India	[71]
Banana, cassava and cow-pea	Improvements in agricultural production and biometric characteristics	Canada and India	[72]
Strawberries	Improvements in the quality of fruits	India	[73]
Strawberries	Improvements of marketable yield and quality of fruits	India	[74]
Sorghum	Development of fruits	Mexico	[75]
Sorghum	Higher development of plants	USA	[76]

**Table 3:** Uses of vermicompost for biocontrol.

Benefited culture	Benefits on crop production	Country	Reference
Grow peppers, tomatoes, and cabbages	Suppression of insect pest populations	USA	[80]
Chickpea (*Cicer arietinum*)	Reductions in plant mortality by decreasing fungal infection	India	[82]
Potato	Decreases in potato scab by increasing the population of *Pseudomonas *	India	[85]
Tomato, marigold, pepper, and cornflower	Higher growth of plants	USA	[86]
Grape and strawberry	Control of nematodes	USA and Turkey	[87]
Cucumbers and tomatoes	Higher resistance against agricultural disease	USA	[88]
Diverse species of plants	Death of phytopathogenic fungi (*Fusarium*)	India	[89]
Cucumbers and tomatoes	Suppression of agricultural plagues	USA	[90, 91]
